# *QuickStats:* Percentage* of Adults^†^ Who Were in Families Having Problems Paying Medical Bills During the Previous 12 Months,^§^ by Race, Hispanic Origin, and Selected Asian^¶^ Subgroups — National Health Interview Survey, United States, 2020−2021**

**DOI:** 10.15585/mmwr.mm7218a7

**Published:** 2023-05-05

**Figure:**
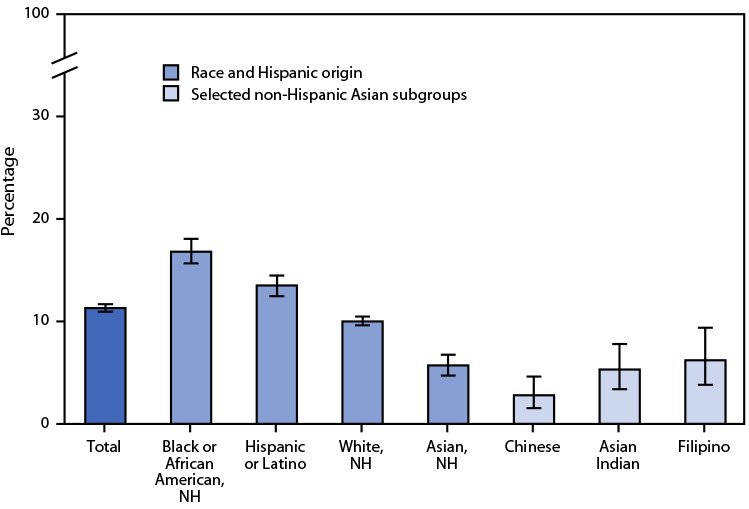
During 2020–2021, the percentage of U.S. adults who were in families having problems paying medical bills during the previous 12 months was 11.3%. Non-Hispanic Asian adults (5.7%) were the least likely to be in families having problems paying medical bills, followed by non-Hispanic White (10.0%), Hispanic or Latino (13.5%), and non-Hispanic Black or African American (16.8%) adults. Among adults within the non-Hispanic Asian origin subgroups shown, those of Chinese origin (2.8%) were less likely to be in families having problems paying medical bills than were adults of Filipino origin (6.2%). Other observed differences were not statistically significant.

**Source:** National Center for Health Statistics, National Health Interview Survey, 2020 and 2021 data. https://www.cdc.gov/nchs/nhis.htm

